# WWL70 attenuates PGE_2_ production derived from 2-arachidonoylglycerol in microglia by ABHD6-independent mechanism

**DOI:** 10.1186/s12974-016-0783-4

**Published:** 2017-01-10

**Authors:** Mikiei Tanaka, Sean Moran, Jie Wen, Kwame Affram, Tinghua Chen, Aviva J. Symes, Yumin Zhang

**Affiliations:** 1 Department of Anatomy, Physiology and Genetics, Uniformed Services University of the Health Sciences, 4301 Jones Bridge Road, Bethesda, MD 20814 USA; 2 Biomedical Instrumentation Center, Uniformed Services University of the Health Sciences, 4301 Jones Bridge Road, Bethesda, MD 20814 USA; 3 Department of Pharmacology and Molecular Therapeutics, Uniformed Services University of the Health Sciences, 4301 Jones Bridge Road, Bethesda, MD 20814 USA; 4 Neuroscience Program, Uniformed Services University of the Health Sciences, 4301 Jones Bridge Road, Bethesda, MD 20814 USA

**Keywords:** Endocannabinoid, 2-AG, Arachidonic acid, ABHD6, COX-2, PGE_2_, PGE_2_-glyceryl ester, PGE synthase, BV2 cells, Microglia

## Abstract

**Background:**

α/β-Hydrolase domain 6 (ABHD6) is one of the major enzymes for endocannabinoid 2-arachidonoylglycerol (2-AG) hydrolysis in microglia cells. Our recent studies have shown that a selective ABHD6 inhibitor WWL70 has anti-inflammatory and neuroprotective effects in animal models of traumatic brain injury and multiple sclerosis. However, the role of ABHD6 in the neuroinflammatory response and the mechanisms by which WWL70 suppresses inflammation has not yet been elucidated in reactive microglia.

**Methods:**

The hydrolytic activity and the levels of 2-AG in BV2 cells were measured by radioactivity assay and liquid chromatography coupled with tandem mass spectrometry (LC-MS/MS). The expression of cyclooxygenase-2 (COX-2) and prostaglandin E_2_ (PGE_2_) synthases in microglia treated with lipopolysaccharide (LPS) with/without WWL70 was determined by western blot and quantitative RT-PCR. The conversion of 2-AG to PGE_2_ or PGE_2_-glyceryl ester (PGE_2_-G) was assessed by enzyme-linked immunoassay (EIA) or LC-MS/MS. The involvement of ABHD6 in PGE_2_ production was assessed using pharmacological inhibitors and small interfering RNA (siRNA). The effect of WWL70 on PGE_2_ biosynthesis activity in the microsome fraction from BV2 cells and experimental autoimmune encephalopathy (EAE) mouse brain was also examined.

**Results:**

We found that WWL70 suppressed PGE_2_ production in LPS-activated microglia via cannabinoid receptor-independent mechanisms, although intracellular levels of 2-AG were elevated by WWL70 treatment. This reduction was not attributable to WWL70 inhibition of ABHD6, given the fact that downregulation of ABHD6 by siRNA or use of KT182, an alternative ABHD6 inhibitor failed to suppress PGE_2_ production. WWL70 attenuated the expression of COX-2 and PGES-1/2 leading to the downregulation of the biosynthetic pathways of PGE_2_ and PGE_2_-G. Moreover, PGE_2_ production from arachidonic acid was reduced in the microsome fraction, indicating that WWL70 also targets PGE_2_ biosynthetic enzymes, which are likely to contribute to the therapeutic mechanisms of WWL70 in the EAE mouse model.

**Conclusions:**

WWL70 is an anti-inflammatory therapeutic agent capable of inhibiting PGE_2_ and PGE_2_-G production, primarily due to its reduction of COX-2 and microsomal PGES-1/2 expression and their PGE_2_ biosynthesis activity in microglia cells, as well as in the EAE mouse brain.

**Electronic supplementary material:**

The online version of this article (doi:10.1186/s12974-016-0783-4) contains supplementary material, which is available to authorized users.

## Background

2-Arachidonoylglycerol (2-AG) and N-arachidonoylethanolamine, also known as anandamide (AEA), are the two principal endogenous ligands for cannabinoid type 1 (CB1) receptor expressed primarily in neurons [1] and the CB2 receptor mostly expressed in microglia and astrocytes, although they are also found in neuronal cells in the brain stem and other regions in the central nervous system (CNS) [2, 3]. A growing body of evidence has indicated that modulation of the endocannabinoid system plays an essential role in ameliorating the development of several neurological diseases such as multiple sclerosis [4, 5], traumatic brain injury [6], and neuropathic pain [7]. Activation of CB1 and/or CB2 receptors contributes to the suppression of inflammation and reduction of glutamate excitotoxicity [8, 9].

In addition to functioning as a full agonist for CB1 and CB2 receptors, 2-AG is also a precursor for arachidonic acid (AA) and its derivative prostaglandins (PGs; PGD_2_, PGE_2_, PGF_2α_, PGI_2_, and TXA_2_) in the brain [10, 11]. Although PGs are lipid mediators involved in cell homeostasis, they also mediate the inflammatory response in a variety of pathological conditions [12]. There is a consensus that AA is mainly provided from diet or derived from glycerophospholipids catalyzed by phospholipase A_2_ [13]. AA is further metabolized to PGs by cyclooxygenases (COXs) and PG synthases. However, recent studies have shown that inhibition or deletion of the primary 2-AG hydrolytic enzyme monoacylglycerol lipase (MAGL) significantly reduces the brain levels of AA and PGs, suggesting that 2-AG plays an essential role in the production of PGs especially in the CNS [14].

The majority of the endocannabinoid-hydrolyzing enzymes belong to the serine hydrolase superfamily [15]. Using a novel technology that detects active serine hydrolases [16], WWL70 was revealed to be a specific inhibitor for α/β-hydrolase domain 6 (ABHD6), a 2-AG hydrolytic enzyme in the brain [17]. The inhibitory property of WWL70 on ABHD6 was further demonstrated by several later studies [18–20]. Although the results from our laboratory and others have shown that only ~4% of 2-AG hydrolysis in the mouse brain and spinal cord is attributable to ABHD6 under physiological conditions [21], ABHD6 contributes significantly to the 2-AG hydrolysis in microglia/macrophages, in which there is no or little expression of MAGL [18, 19]. Recently, we have found that WWL70 exerts therapeutic effects in mouse models of traumatic brain injury [22] and experimental autoimmune encephalomyelitis (EAE) [23] through attenuating microglial activation, COX-2 expression, and the impaired function of the blood-brain barrier. In addition, WWL123, a derivative of WWL70 with high permeability to the blood-brain barrier, was also protective in a mouse model of epilepsy [24]. However, the exact mechanism by which inhibition of ABHD6 results in the reduction of inflammatory response remains elusive.

In this study, we found that treatment with WWL70 substantially reduced PG production in BV2 and primary microglia cells. Surprisingly, this reduction was not due to inhibition of ABHD6 by WWL70 but was attributable to its interference with the metabolic pathway from AA to PGE_2_. Moreover, we found that WWL70 inhibited microsomal PGE_2_ synthesis activity, which is the primary cause for PGE_2_ reduction. Thus, we report here a novel inhibitory action of WWL70 on microglia by modulating PGE_2_ biosynthesis activity.

## Methods

### Cell culture

BV2 microglial cells [25] were cultured in complete DMEM containing 5% heat-inactivated fetal bovine serum (Life Technologies, Grand Island, NY) under humidified 5% CO_2_ environment. The cells were maintained by medium change every other day. One day prior to experiment, cells were plated on 24-well plates to reach 90% confluence at the beginning of experiment.

For primary microglia culture, Sprague–Dawley rats were purchased from Charles River Laboratories (Frederick, MD) and housed in the animal facility of the Uniformed Services University of the Health Sciences (USUHS). All animal protocols were approved by the USUHS Institutional Animal Care and Use Committee. P2 Sprague–Dawley rat pups (male and female) were sacrificed and their brains removed. Mixed glial cultures were then prepared from the cortices after careful removal of the meninges and brain stem. The cortices were then triturated by serial trituration with a 10-ml pipette, 18-G needle (3×), 22-G needle (3×), and finally, a 25-G needle (2×) in culture medium (DMEM containing 10% fetal bovine serum, 1% glutamax, and 1% antibiotic-antimycotic). After trituration, the suspension was filtered using a 70-micron mesh and then pelleted at 168×*g* for 10 min at room temperature. Cells were resuspended in culture medium, seeded into T75 flasks, and incubated in a CO_2_ incubator. The medium was replaced every 2–3 days. After 14 or 15 days in culture, primary microglia were harvested by differential shaking on an orbital shaker for 1 h at 200 rpm in a CO_2_ incubator. The medium, containing the detached microglia, was collected and centrifuged at 671×*g* for 5 min at room temperature. Cells were then resuspended with DMEM containing 10% normal horse serum, 1% glutamax, and 1% streptomycin/penicillin and transferred to uncoated plates at a density of 2.5 × 10^5^ cells/mL.

### Reagents

KT182, an ABHD6 inhibitor, and HT-01, the activity-based protein profiling (ABPP) probe specific for ABHD6, were kindly provided by Drs. Hsu and Cravatt [26]. 2-Arachidonoylglycerol [glycerol-1,2,3-^3^H] was from American Radiolabeled Chemicals Inc. (Saint Louis, MO). Cyclooxygenase inhibitor assay kits including COX inhibitor and recombinant COX-1 or COX-2 activity assay kit were from Cayman Chemical (Ann Arbor, MI). siRNA (FlexiTube Mm_abhd6_3 and Allstars Negative Control) and HiPerfect transfection reagent were from QIAGEN (Valencia, CA). WWL70, methyl arachidonyl fluorophosphonate (MAFP), SR141716 (SR1), SR144528 (SR2), 2-AG, 2-AG-d_8_, and AA were purchased from Cayman Chemical. Other reagents including lipopolysaccharide (LPS) were purchased from Sigma-Aldrich (St. Louis, MO).

### PGE_2_ enzyme immunoassay

A multi-well cell culture plate was prepared 1 or 2 days prior to the test. The cell culture medium was replaced with pre-warmed medium containing the ABHD6 inhibitor WWL70 (10 μM) and incubated for 15 min. The cells were treated with 10 μM of 2-AG for 15 min, followed by addition of 100 ng/ml LPS for BV2, or 2 ng/ml LPS for primary microglia. After incubation for 18 h, the culture medium was collected. Before addition to the enzyme-linked immunoassay (EIA), the medium was centrifuged at 5000 rpm for 2 min with a table top centrifuge to exclude residual cells. To determine the role of WWL70 on PGE_2_ production in vivo, EAE was induced by subcutaneous injection of myelin oligodendrocyte glycoprotein peptide 35–55 (MOG_35–55_) in 8-week-old female C57BL/6 mice, and the clinical score was assessed as we reported recently [23]. WWL70 (10 mg/kg, i.p.) was given starting at the disease onset and then once a day until the end of the test. The mouse forebrain at 3 weeks post-immunization was dissected and kept frozen at −80 °C until use. The forebrain tissue was homogenized with one-fifth volume of 0.02% trifluoroacetic acid (TFA) and one volume of acetonitrile using a Potter homogenizer at 4 °C. Homogenate was dispersed completely in 2 ml of acetonitrile by vortex and stored at 4 °C overnight. Debris was excluded by centrifugation at ×2000*g* for 5 min, and then the supernatant was evaporated under the nitrogen gas streaming in a water bath (approximately 35 °C). After reconstitution with the EIA buffer, the levels of PGE_2_ were measured following the manufacturer’s protocol (Cayman Chemical, Ann Arbor, MI).

### 2-AG hydrolysis activity assay

Hydrolysis activity of 2-AG was assessed as previously described [27]. For hydrolysis activity in intact cells, BV2 cells in a 24-well plate were pre-incubated with or without inhibitor for 30 min at 37 °C, followed by washing with pre-warmed DMEM plus 0.15% fatty acid-free BSA. The cells were incubated with 0.2 ml of 10 μM radiolabeled ^3^[H]-2-AG (33 nCi) in DMEM containing 0.15% fatty acid-free BSA. After 2 min of incubation, the reaction was terminated by mixing with 0.4 ml chilled methanol. The cell extract was transferred to a silanized glass tube, followed by mixing with 0.4 ml of chloroform by brief vortexing. The mixture was centrifuged at ×3000*g* for 5 min to separate the aqueous and organic phases. Aliquot of the aqueous phase was mixed with scintillation cocktail and measured in a scintillation counter LS6500 (Beckman Coulter, Brea, CA). For hydrolysis activity in membrane fraction, BV2 cell homogenate was centrifuged at ×1400*g* for 5 min to remove the debris, then ultracentrifuged at ×100,000*g* at 4 °C for 30 min. The pellet was resuspended with PBS, and protein concentration was determined by DC protein assay kits using BSA as a standard (Bio-Rad). Four hundred microliters of the membrane fraction (20 μg) was pre-incubated in a silanized glass tube with or without 10 μM of WWL70 for 5 min at 37 °C, then mixed with 100 μl of 10 μM ^3^[H]-2-AG (33 nCi) in PBS containing 0.15% fatty acid-free BSA. After 2 min of incubation, the reaction mixture was mixed with chilled 2 ml methanol/chloroform (1:1). Radioactivity in the aqueous phase was measured as described above.

### Activity-based protein profiling and western blotting

One milligram per milliliter of the membrane fraction prepared as above was pre-incubated with WWL70 at indicated concentrations for 10 min at room temperature, then mixed with 1 μM HT-01, a serine hydrolase probe that specifically recognizes active ABHD6 enzymes [26], at 37 °C for 40 min. The reaction mixture was added to the SDS-PAGE sample buffer and heated at 95 °C for 5 min. Approximately 10 μg of the protein was loaded on SDS-PAGE. The gel was scanned with a fluorescence imager, Fuji FLA-5100 (Fujifilm, Edison, NJ) with an excitation wavelength at 473 nm using FITC mode. The intensity of the fluorescent band is proportional to the amount of active ABHD6. Subsequently, the gel was transferred onto PVDF membrane for western blotting with anti-calnexin as a loading control for the membrane fraction.

For western blotting, cell lysate or microsome fraction was prepared with RIPA buffer containing 150 mM NaCl, 50 mM Tris-HCl (pH 8.0), 1% Triton X-100, 0.5% Na deoxycholate, 0.1% SDS, 1 mM EDTA, 1 mM EGTA, 1 mM Na_3_VO_4_, 1 mM β-glycerophosphate, and protease inhibitor cocktail (Roche Applied Sciences) for 5 min on ice, followed by centrifugation at ×12,000*g* for 5 min at 4 °C to remove the debris. Transferred PVDF membrane was pre-incubated with 5% BSA in PBS + 0.05% Tween-20 (PBST) for 30 min, then incubated with antibodies against β-actin (AC-74, Sigma-Aldrich) at 1:1000, calnexin (T-40, Santa Cruz) at 1:1000, microsomal prostaglandin E2 synthase (mPGES)-1 (#160140, Cayman Chemical) at 1:500, mPGES-2 (#160145, Cayman Chemical) at 1:500, and COX-2 (#160106, Cayman Chemical) at 1:250 in PBST at 4 °C overnight. The transferred membrane was reacted with a secondary antibody conjugated with horseradish peroxidase (Bio-Rad) at 1:2500 for 2 h, followed by visualization with ECL reagent (Thermo Scientific) using the LAS3000 imager (Fujifilm). The relative intensity of COX-2 to β-actin was quantified using ImageJ software (National Institutes of Health), and the fold change compared to the LPS treatment alone was presented.

### LC-MS/MS analysis for 2-AG

Sample preparation for 2-AG quantification was carried out based on a method published previously [28]. BV2 cells (90% confluence) in 10-cm dishes were treated with WWL70 (10 μM) or MAFP (10 μM) for 1 h. After rinsing with PBS once, the cells were collected by centrifugation at ×5000*g* for 2 min. The pellet was suspended with 0.1 ml of 0.02% TFA and 1 nmole of 2-AG-d_8_ by pipetting and dispersed in 4 ml of acetonitrile in a silanized glass tube to precipitate the debris overnight at −20 °C. The supernatant after centrifuged at ×5000*g* for 5 min was transferred to a new glass tube and evaporated under a nitrogen gas stream in a mild hot water bath (approximately 35 °C). 2-AG was resuspended with 0.1 ml of acetonitrile and stored at −80 °C until mass analysis.

An HPLC system (1200 Series, Agilent Technologies, Santa Clara, CA) was used with a reverse phase guard column (Wide Pore C18 (ODS), 4 × 2 mm ID; Phenomenex, Torrance, CA), and the column (Sephasil Peptide C18, 5 u, ST, 100 × 4.6 mm ID; Pharmacia Biotech (Amersham), Piscataway, NJ) was maintained at 40 °C. The mobile phase was composed of solvent A: 0.2% formic acid in water, and solvent B: 0.2% formic acid in methanol; the following gradient was used: 62% A/38% B isocratic for 30 s, ramp to 90% B in 60 s, isocratic at 90% B for 18.5 min, ramp back to 62% A/38% B in 60 s, and re-equilibrate at 62% A/38% B for 8 min. The flow rate was 0.4 ml/min. The HPLC output was directed into the TurboV electrospray ionization (ESI) source of a Q-Trap 4000 mass spectrometer (AB Sciex, Framingham, MA). The injection volume was 20 μl. LC-MS/MS analysis was performed in a positive mode with the ion source temperature of 600 °C, a spray voltage of 5.5 kV, and a declustering potential of 45 V. Multiple reaction monitoring (MRM) was performed on the transitions *m*/*z* 379.5→287.5 for 2-AG and 386.5→293.5 for 2-AG-d_8_. Calibration curves of the ratio of analyte (2-AG) to internal standard (2-AG-d_8_) peak areas vs. concentration ratio (at least seven different 2-AG concentrations at a single fixed 2-AG-d_8_ concentration) were generated by linear least squares fitting and used to calculate the 2-AG concentrations in the samples (converting the observed *y*-value (peak area ratio) of the samples into *x*-values (concentration ratios with a fixed internal standard concentration).

### LC-MS/MS for PGE_2_ and PGE_2_-G

The method for LCMS analysis of PGE_2_ and PGE_2_-glycerol in positive ion mode was derived from a different previously published procedure [29]. BV2 cells pretreated with or without 0.1 μg/ml of LPS for 8 h were rinsed once with pre-warmed serum-free medium (Opti-MEM, Thermo Fisher Scientific), then incubated with Opti-MEM containing 10 μM 2-AG, 10 μM WWL70, or both for 30 min in CO_2_ incubator. The medium was collected and centrifuged at ×1000*g* for 5 min to remove floating cells and stored at −80 °C until use. One milliliter of medium was mixed with 1.4 ng PGE_2_-d4 and 0.5 ng PGE_2_-G-d5 (Cayman Chemical) and acidified by glacial acetic acid to 1% at final concentration. It was loaded to a solid-phase extraction column (Oasis HLB 1 cc, Waters Corporation, Milford, MA) equilibrated with 0.5% acetic acid and methanol. After washing with 0.5% acetic acid followed by 0.5% acetic acid with 15% methanol, PGs were eluted with 1.5 ml of methanol. After evaporation in nitrogen streaming under approximately 30 °C, the PGs were reconstituted with acetonitrile/water (1:2).

The same LCMS system described above was used with a Higgins Analytical TARGA C18 3 μm 2.1 × 100 mm column (Nest Group Inc., Southborough, MA). The HPLC gradient was as follows: the column was equilibrated at 80% 5 mM ammonium formate in water (buffer A)/20% 4:1 acetonitrile/water with 5 mM ammonium formate (buffer B); after sample injection, the gradient for elution of analytes went from 20 to 80% buffer B in 15 min. The column was re-equilibrated by ramping to 100% buffer B in 1 min, holding at 100% buffer B for 2 min to flush, ramping back down to the initial HPLC buffer composition (80%/20% A:B) in 2 min, and holding at the initial buffer composition for 8 min before the next injection. The flow rate was 0.2 mL/min, the column temperature was 40 °C, and the injection volume was 25 μL. The ion source temperature was 550 °C, and the declustering potential was 40 V. Since the HPLC buffer contained ammonium ions, the precursor ions for PGE_2_ and PGE_2_-glycerol were ammonium adducts (M + 18). The following MRM transitions were used: for PGE_2_, precursor ion mass 370.3 Da to fragment ion mass 335.2 Da (collision energy (CE) 12.5 V) and 317.1 Da (CE 15 V); for d4-PGE_2_, 374.3 to 339.2 Da (CE 12.5 V) and 321.3 Da (CE 15 V); for PGE_2_-glycerol, 444.4 to 409.2 Da (CE 12.5 V) and 299.2 Da (CE 26 V); and for d5-PGE_2_-glycerol, 449.4 to 414.1 Da (CE 12.5 V) and 299.2 Da (CE 26 V).

### qRT-PCR

Total RNA from BV2 cells or primary microglia treated with reagents for 8 h was isolated using TRIzol (Life Technologies) according to the manufacturer’s protocol. Five hundred nanograms of RNA were used for cDNA synthesis using MAXIMA First Strand cDNA synthesis kit with dsDNAse (Thermo Fisher Scientific) following the manufacturer’s protocol. The Cq value of negative control without reverse transcription was >40 due to the treatment with DNase. Complementary DNA from 10 ng RNA was employed for qPCR in the presence of 250 nM of gene-specific primers (mPGES-1 forward, 5′-TGTCCAAATCCTGTCTTCCA-3′, reverse, 5′-GGTTCTGGAGCACACCCTAT-3′; mPGES-2 forward, 5′-GAAATGGCTGCAGAATTGAA-3′, reverse, 5′-AAGGAGAATGGTGCTCCAAG-3′; COX-2 forward, 5′-GAAATGGCTGCAGAATTGAA-3′, reverse, 5′-AAGGAGAATGGTGCTCCAAG-3′; ABHD6 forward, 5′-CATTCCAATCCTGGCATTTGTTG-3′, reverse, 5′-ATGGTGTGCGTAGCGAACTT-3′; and GAPDH forward, 5′-AGGTCGGTGTGAACGGATTTG-3′, reverse, 5′-TGTAGACCATGTAGTTGAGGTCA-3′) using Power SYBR Green PCR master mix (Life Technologies) in 12 μl reaction mixture. Thermal cycling condition was 95 °C for 10 min, followed by 40 cycles of 95 °C × 15 s and 60 °C × 60 s performed by LightCycler 480 II (Roche Life Science, Indianapolis, IN). Gene-specific PCR amplification was confirmed by the melting curve profile using LightCycler 480 system program showing 80 to 85 °C of single melting temperature for all the genes tested. GAPDH was used as a reference gene because its Cq value was constant among the cells tested (Cq value is 15.7 ± 0.1, mean ± SD). The PCR amplification linearity was confirmed using serial diluted cDNA showing the consistent amplification in the concentration range tested (*r* ^2^ > 0.998).

### siRNA transfection

BV2 cells in a 24-well plate (50 to 70% confluent) were transfected with siRNA for the mouse ABHD6 gene (Mm_abhd6_3, QIAGEN) or the negative control using HiPerfect transfection reagent according to the manufacturer’s protocol. Briefly, 37.5 ng siRNA was mixed with 100 μl DMEM without FBS, then added 6 μl of the transfection reagent and incubated for 10 min at room temperature. The siRNA complex suspension was added to the cells drop-wise. Total RNA was isolated from the cells after 1 day of incubation and qRT-PCR was performed to examine the expression of ABHD6 and GAPDH. Otherwise, the transfected cells after 2 days of incubation were used to prepare membrane fraction for ABPP to assess ABHD6 hydrolytic activity, followed by western blot with the calnexin antibody as a loading control.

### Enzyme activity assay

The COX assay was carried out using the COX inhibitor screening assay kit from Cayman Chemical. Briefly, ovine recombinant COX-1 or human recombinant COX-2 was pre-incubated in 0.1 M Tris-HCl (pH 8.0) and EDTA (1 mM) buffer with WWL70 (10 μM), SC-560 (0.33 μM), or Dup-697 (0.3 μM) for 5 min at 27 °C. Then, 10 μM of AA was added and incubated for 1 min at 27 °C. The reaction was stopped by adding 1 N HCl. Stannous acid was then added to convert PGH_2_ to the stable product PGF_2α_. The resultant mixture was applied to a prostaglandin EIA to measure the total amount of prostaglandins including PGE_2_ and PGF_2α_. The PGE_2_ biosynthesis assay was performed using microsomes derived from BV2 cells treated with LPS for 8 h or brain tissue from EAE mouse at day 21 post-immunization [23]. BV2 cells were suspended with 0.1 M potassium phosphate (pH 7.4), 0.25 M sucrose, 1 mM β-glycerophosphate, 1 mM sodium vanadate, 1 μM MAFP, and protease inhibitor cocktail (Roche) and disrupted by sonication for 15 s, while the brain tissue was homogenized with a Potter homogenizer with the potassium phosphate buffer at 4 °C. These homogenates were centrifuged at ×1400*g* for 10 min at 4 °C to remove the debris, followed by further centrifugation at ×10,000*g* for 10 min at 4 °C. The supernatant was ultracentrifuged at ×170,000*g* for 1 h at 4 °C to precipitate the microsome fraction. The pellet was suspended with potassium phosphate buffer containing glutathione (2.5 mM) by brief sonication. One hundred micrograms per milliliter of BV2 microsomes was pre-incubated with WWL70, MK886 (3 μM), SC-560 (1 μM), or NS398 (10 μM) for 5 min at 23 °C, then mixed with 10 μM of AA for 1 min at 23 °C. 500μg/ml brain microsomes were incubated with 10 μM of AA for 2 min at 23 °C. The reaction was stopped by mixing with stannous acid (5 mg/ml in 0.1 N HCl) to deactivate the enzyme and convert intermediate PGH_2_ to PGF_2α_, followed by the measurement of PGE_2_ concentration by EIA as described above. The activity was determined after subtraction with the amount of PGE_2_ in the microsome fraction incubated without substrate.

### Statistics

All data are expressed as mean ± SD. The GraphPad Prism 7 (GraphPad Software Inc., San Diego, CA) was used for statistical analysis. The statistical comparison among the drug-treated groups (different drugs or the same drug at various concentrations) was performed using one-way ANOVA followed by Turkey’s test; while in experiments with both LPS, LPS + 2-AG, or LPS + AA groups, two-way ANOVA was performed. An unpaired *t* test was used for data comparison between two groups. Statistical significance was set at *P* < 0.05, with single, double, and triple asterisks to denote *p* < 0.05, 0.01, and 0.001, respectively.

## Results

### WWL70 inhibited 2-AG hydrolysis and elevated 2-AG level in BV2 cells via inhibition of ABHD6

**Fig. 1:**
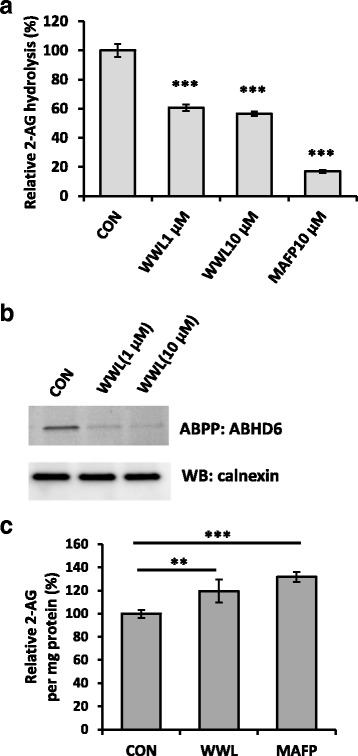
Inhibition of ABHD6 by WWL70 increased the 2-AG levels in BV2 cells. **a** BV2 cells were treated with WWL70 or MAFP for 30 min and then incubated with ^3^[H]-2-AG at 37 °C for 2 min. Cell lysates were mixed with MeOH/CHCl_3_ to separate the organic from aqueous phase. The amount of ^3^[H]-glycerol, the product of 2-AG hydrolysis, was measured in the aqueous phase. Inhibitors were added at either 1 or 10 μM. Data are represented as mean ± SD (*n* = 3). *Triple asterisks* denote *p* < 0.001 compared to the drug-treated groups. **b** The membrane fraction from BV2 cells was incubated with WWL70 for 10 min, reacted with HT-01 probe (1 μM) at 37 °C for 40 min, and then applied to SDS-PAGE. The gel was scanned to detect active ABHD6, followed by western blotting with an anti-calnexin antibody. **c** BV2 cells were incubated with 10 μM of WWL70 or MAFP for 1 h before harvest. Cell lipids were extracted with acetonitrile plus 0.02% TFA together with 2-AG-d_8_ as an internal standard. 2-AG was identified and quantified with LC-MS/MS based on the ratio to the internal standard. Data are represented as mean ± SD (*n* = 6). *Double* and *triple asterisks* denote *p* < 0.01 and 0.001, respectively, compared to control

2-AG hydrolysis to AA in BV2 cell membrane was confirmed by deuterated AA generation using LC-MS/MS (Additional file 1: Figure S1). Because AA can be rapidly metabolized, we measured the simultaneously produced glycerol derived from 2-AG using radioactive assay. In the presence of WWL70, 2-AG hydrolysis in BV2 cells was decreased by 40%, while the general serine hydrolase inhibitor, methyl arachidonyl fluorophosphonate (MAFP) [30], almost completely inhibited the hydrolytic activity (Fig. 1a). This result is consistent with a previous report [19] indicating that ABHD6 is one of the main 2-AG hydrolytic enzymes in BV2 cells. ABPP is a recently developed technology to detect serine hydrolases using a probe that specifically binds to the active enzymes but not the inactive or the inhibitor bound forms [15]. Using the HT-01 probe, we found that WWL70 at 1 μM and 10 μM dramatically reduced the band intensity of ABHD6 (Fig. 1b). Next, we examined whether inhibition of ABHD6 could alter the intracellular levels of 2-AG with LC-MS/MS. At 1 h after WWL70 (10 μM) treatment, 2-AG was increased by 20% compared to untreated cells (Fig. 1c). Treatment with MAFP (10 μM) also significantly increased 2-AG levels (Fig. 1c).

### WWL70 reduced PGE_2_ production in LPS-activated BV2 cells

**Fig. 2:**
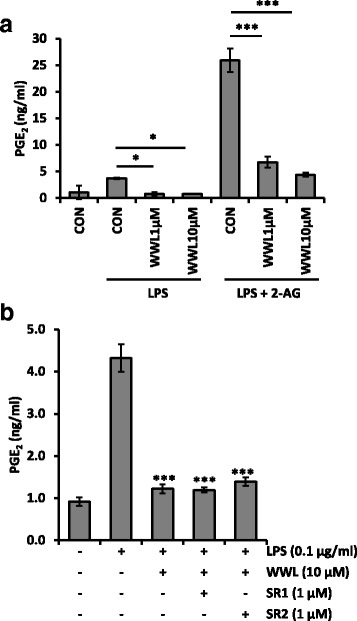
WWL70 blocked PGE_2_ production independently on CB receptor signaling. **a** BV2 cells were incubated with WWL70 for 15 min, before addition of 2-AG (10 μM) for 15 min, and subsequent addition of LPS (100 ng/ml). After 18 h, the culture medium was tested for PGE_2_ by EIA. **b** BV2 cells were treated with LPS, WWL70 with/without CB1R antagonist SR1 (SR141716A), or CB2R antagonist SR2 (SR144528) for 18 h before the culture medium was assayed for PGE_2_ content. There were no significant differences in the amount of PGE_2_ with or without antagonist. Representative experiment, mean ± SD (*n* = 3), out of three similar independent experiments. *Single* and *triple asterisks* denote *p* < 0.05 and 0.001, respectively, compared to the drug-treated groups

It has been reported that the hydrolysis of 2-AG by MAGL is one of the major sources of AA and its derivative PGs in the mouse brain [10, 14]. Because ABHD6, but not MAGL is the primary 2-AG hydrolytic enzyme in BV2 cells [18, 19], we tested the possibility that WWL70 could reduce PGE_2_ production in BV2 cells. Treatment with LPS (100 ng/ml) for 18 h increased PGE_2_ production, and this increase was completely blocked by WWL70 at either 1 or 10 μM (Fig. 2a). To further determine if PGE_2_ production is affected by 2-AG metabolism, cells were incubated for 18 h in the presence of 2-AG and LPS. Exogenous addition of 2-AG together with LPS caused a sevenfold increase of PGE_2_ production compared to cells incubated with LPS alone, suggesting that the increased PGE_2_ was derived from 2-AG. In the presence of 2-AG, the production of PGE_2_ was reduced substantially by 1 and 10 μM WWL70 (Fig. 2a).

To determine whether PGE_2_ reduction by WWL70 is mediated by cannabinoid receptors, cells were co-incubated with the CB1 receptor antagonist SR141716A (SR1) or the CB2 receptor antagonist SR144528 (SR2). Neither CB1 nor CB2 antagonist reversed the PGE_2_ reduction by WWL70, suggesting that cannabinoid receptor signaling was not involved in the inhibitory effect of WWL70 (Fig. 2b).

### The expression levels of COX-2 and mPGES in LPS-activated BV2 cells were reduced by WWL70

**Fig. 3:**
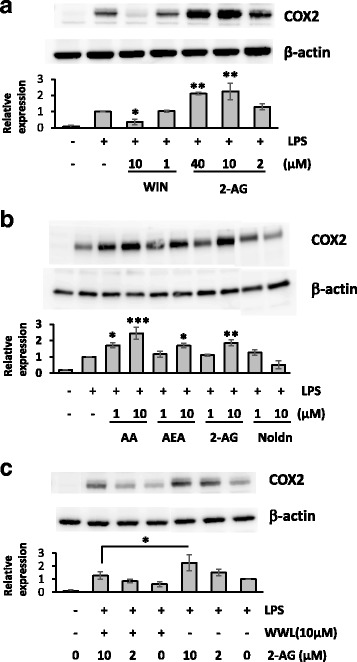
WWL70 suppressed COX-2 expression. Western blots of BV2 cell lysates with different treatment protocols, showing COX-2 expression with β-actin controls. The blots shown here are representative of three independent experiments. **a** BV2 cells were incubated with synthetic CB receptor agonist, WIN55,212-2, or 2-AG for 15 min, before addition of LPS (0.1 μg/ml) for 18 h. *Single* and *double asterisks* denote *p* < 0.05 and 0.01, respectively, compared to the LPS treatment alone (mean ± SD, *n* = 3). **b** BV2 cells were treated with AA or endocannabinoids at the indicated concentrations for 15 min followed by incubation with LPS for 18 h. *Single*, *double*, and *triple asterisks* denote *p* < 0.05, 0.01, and 0.001, respectively, when compared to the LPS treatment alone (mean ± SD, *n* = 3). **c** BV2 cells were treated with WWL70 for 15 min, followed by the addition of 2-AG for a further 15 min, before addition of LPS for 18 h. Relative COX-2 expression levels to β-actin were shown under each blot after normalizing with the LPS-treated conditions. *Single asterisk* denotes *p* < 0.05 (mean ± SD, *n* = 3). WIN refers to WIN55,212-2, and Noldn refers to 2-arachidonyl glyceryl ether

**Fig. 4:**
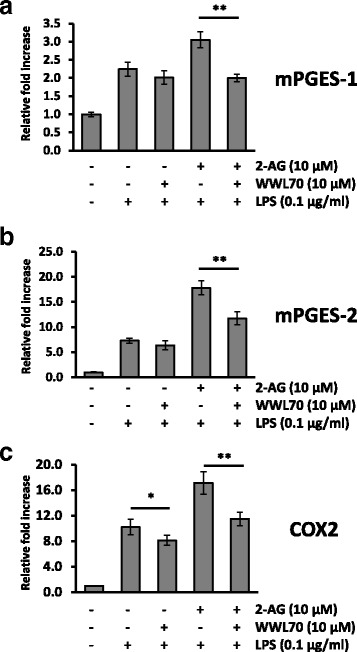
WWL70 suppressed microsomal PGESs and COX-2 mRNA expression. qRT-PCR of mPGES-1 (**a**), mPGES-2 (**b**), or COX-2 (**c**) mRNA isolated from BV2 cells treated with WWL70 (10 μM) for 15 min, before addition of 2-AG (10 μM) for 15 min, and then LPS (0.1 μg/ml) for 18 h. Data are represented as relative expression to control condition after normalization with GAPDH (mean ± SD, *n* = 4). *Single* and *double asterisks* denote *p* < 0.05 and 0.01, respectively

Since COX-2 is involved in the metabolism of AA to PGE_2_, the potentiation of 2-AG on PGE_2_ production in LPS-activated BV2 cells might be due to the increased expression of COX-2. To test this possibility, BV2 cells were treated with LPS for 18 h in the presence of different concentrations of 2-AG. Expression of COX-2 was induced by LPS and further potentiated by 2-AG (Fig. 3a). However, the CB1 and CB2 cannabinoid receptor agonist WIN55,212-2 dramatically suppressed the expression of COX-2. This result suggests that the expression of COX-2 is differentially regulated by 2-AG and the other synthetic cannabinoid receptor agonists. To determine the action of other endocannabinoid (eCB) ligands, AEA, which activates either CB receptors with comparable efficiency to 2-AG and is hydrolyzed to AA by fatty acid amide hydrolase, was also tested in LPS-treated BV2 cells and shown to upregulate the expression of COX-2. Addition of AA also potentiated the expression of COX-2, whereas the Noladin ether, which is an unhydrolyzable eCB ligand, did not show any increase (Fig. 3b). These results suggest that COX-2 upregulation by eCBs was dependent on the production of AA from their hydrolysis. In line with this notion, treatment with WWL70 reduced the increase of COX-2 by 2-AG (Fig. 3c), suggesting that the reduced COX-2 expression was likely due to the limited production of AA. Consistently, the enhanced mRNA expression of mPGES-1 and mPGES-2 (Fig. 4a, b) by LPS was also potentiated by 2-AG and reduced by WWL70 similar to that of COX-2 (Fig. 4c).

### PGE_2_ reduction by WWL70 was independent of ABHD6 inhibition

**Fig. 5:**
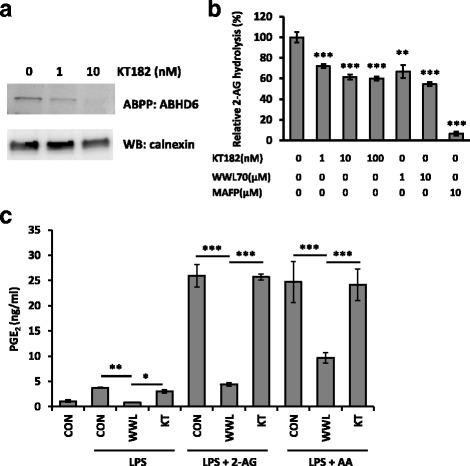
The highly selective ABHD6 inhibitor KT182 did not reduce PGE_2_ production. **a** Activity-based protein profiling with the ABHD6-specific probe HT-01 shows reduced ABHD6 activity in the membrane fraction of BV2 cells in the presence of KT182. **b** Using membrane fraction from BV2 cells, treatment with KT182, WWL70, or MAFP for 5 min showed decreased hydrolysis of 2-AG by quantifying released radioactive glycerol as described in Fig. 1. Relative 2-AG hydrolysis as compared to the non-treated group are shown (mean ± SD; *n* = 3). **c** BV2 cells were incubated with DMEM plus WWL70 (10 μM) or KT182 (100 nM) for 15 min, before addition of 10 μM 2-AG or AA for 15 min, followed by addition of LPS (100 ng/ml) for 18 h. Culture medium was assayed for PGE_2_ by EIA (mean ± SD, *n* = 3). *Single*, *double*, and *triple asterisks* denote *p* < 0.05, 0.01, and 0.001, respectively

**Fig. 6:**
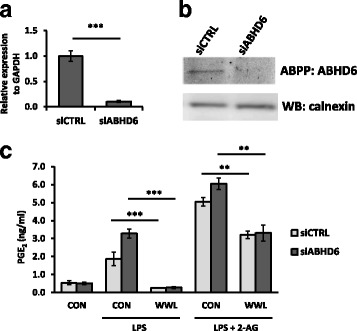
ABHD6 siRNA knockdown did not reduce PGE_2_ production. **a** qRT-PCR of ABHD6 transcript normalized to GAPDH, 24 h after transfection with ABHD6-specific siRNA. **b** Membrane fraction prepared from the transfected cells after 48 h transfection was reacted with the ABHD6-specific HT-01 probe for 30 min to detect active ABHD6 enzyme, followed by western blotting of calnexin as a loading control. **c** Cell cultures after 48 h transfection were replaced with fresh medium with LPS (0.1 μg/ml), WWL70 (10 μM), or 2-AG (10 μM). The culture medium was collected after 18 h incubation and employed PGE_2_ EIA. Data are analyzed with two-way ANOVA and represented as mean ± SD (*n* = 3). *Double* or *triple asterisks* denote *p* < 0.01 or 0.001, respectively

To further examine whether pharmacological inhibition of ABHD6 leads to the reduced PGE_2_ levels, a recently developed ABHD6 inhibitor KT182 was tested. KT182 is a derivative of piperidyl-1,2,3-triazole urea inhibitors with high potency and selectivity to target ABHD6 [26]. Activity-based protein profiling using KT182-treated BV2 membrane fraction showed a strong reduction in active ABHD6 at 1 and 10 nM of KT182 (Fig. 5a), which is consistent with a previously published report that the IC_50_ of this inhibitor is less than 5 nM [26]. The inhibition of 2-AG hydrolysis activity by KT182 in the BV2 membrane fraction was similar to that of WWL70 (Fig. 5b). However, unlike WWL70 which reduced the production of PGE_2_ in LPS-activated BV2 cells, PGE_2_ production was not affected by KT182 in the presence or absence of either 2-AG or AA (Fig. 5c). These data suggest that the inhibition of PGE_2_ production by WWL70 might be independent of ABHD6 inhibition. Next, we tested whether ABHD6 knockdown could affect PGE_2_ production. 24 h after siRNA transfection, the mRNA levels of ABHD6 were reduced by approximately 80% compared to the control siRNA-transfected cells (Fig. 6a). The activity of ABHD6 in BV2 cells after 2 days siRNA transfection was also dramatically reduced (Fig. 6b). Different from the results with WWL70, but consistent with those with KT182, knockdown of ABHD6 did not decrease PGE_2_ production in BV2 cells treated with LPS in the absence or presence of 2-AG. On the other hand, the production of PGE_2_ was slightly increased in siABHD6 cells possibly due to the differences in cell population after transfection. The reduction of PGE_2_ by WWL70 was similar in both control and ABHD6 siRNA-transfected cells (Fig. 6c). These results suggest that the inhibition of PGE_2_ production by WWL70 is independent of its inhibition of ABHD6. Rather, the inhibitory action of WWL70 is likely due to its interference with the biosynthetic pathway from AA to PGE_2_, in which COXs and PGE_2_ synthases are involved.

### WWL70 inhibited PGE_2_ synthesis

**Fig. 7:**
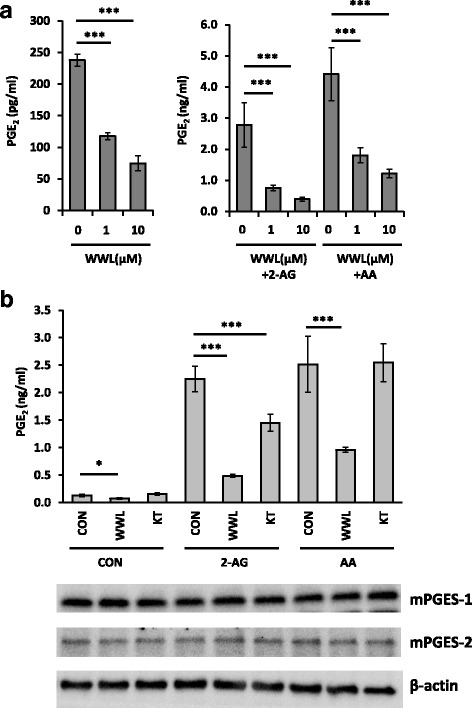
WWL70 inhibited PGE_2_ production in LPS-activated BV2 cells. Cells were treated with LPS (0.1 μg/ml) for 18 h to activate PGE_2_ biosynthesis before replacement with fresh DMEM containing (**a**) 10 μM 2-AG or AA and WWL70 (1 or 10 μM) or (**b**) 10 μM 2-AG or AA and WWL70 (10 μM) or KT182 (100 nM). The culture medium after 30 min incubation was employed PGE_2_ EIA. Using the microsome fraction, mPGES-1, mPGES-2, and β-actin protein expression after 30 min incubation with different treatment conditions was examined by western blot as shown below the EIA results. Data are represented as mean ± SD (*n* = 3). *Single* and *triple asterisks* denote *p* < 0.05 and 0.001, respectively

We examined whether WWL70 can modulate PGE_2_ production by inhibition of the activity of PGE_2_ biosynthetic enzymes, rather than through suppression of COX-2 and PGES expression. Eighteen hours after treatment with LPS to activate the PGE_2_ biosynthetic pathway, the medium was replaced with fresh medium containing WWL70 with or without either 2-AG or AA, and then incubated for a short time period (30 min). The production of PGE_2_ was reduced by WWL70 in the absence or the presence of 2-AG or AA with a similar dose-response (Fig. 7a). KT182 treatment did not alter the amount of PGE_2_ generated, except for the co-treatment with 2-AG (Fig. 7b). Following the 30-min drug treatment, the expression of mPGES-1 and mPGES-2 was not changed (see western blot images in Fig. 7b). These results suggest that the enzymatic activity of COX-2 or PGES is inhibited by WWL70, but not by KT182.

### WWL70 inhibited PGE_2_-G production

**Fig. 8:**
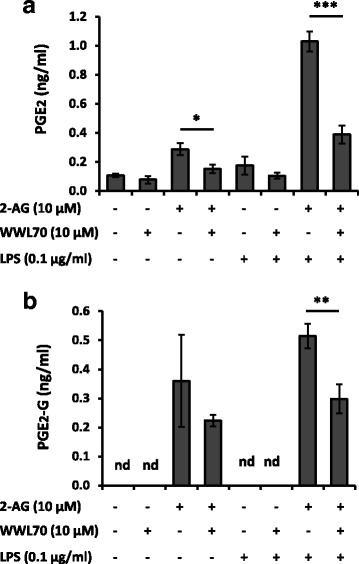
Determination of WWL70 inhibitory effect on PGE_2_ and PGE_2_-G productions using LC/MS/MS. BV2 cells treated with LPS (0.1 μg/ml) for 8 h were replaced with fresh non-serum medium containing 2-AG with/without WWL70 and incubated for 30 min. PGs in the medium was extracted with SPE, then employed LC-MS/MS together with internal standards PGE_2_-d_4_ and PGE_2_-G-d_5_ to determine the quantity of PGE_2_ (**a**) and PGE_2_-G (**b**). nd denotes not detected. Data are represented as mean ± SD (*n* = 3 or 4)

We further examined the possibility that WWL70 inhibits PGE_2_-G production, since the biosynthetic pathways for synthesis of PGE_2_-G and PGE_2_ overlap. BV2 cells activated by LPS were subsequentially incubated with non-serum containing medium with 2-AG or WWL70 for 30 min. The PGs in the medium were analyzed with LC-MS/MS. Consistent with previous results, PGE_2_ production was increased by LPS treatment and boosted in the presence of 2-AG, whereas WWL70 decreased PGE_2_ production (Fig. 8a). PGE_2_-G was detected only under the conditions in the presence of 2-AG, but not in the absence of 2-AG, indicating that the PGE_2_-G was derived from 2-AG (Fig. 8b). Treatment with WWL70 reduced the PGE_2_-G significantly in the cells treated with LPS and 2-AG (Fig. 8b).

### WWL70 targets PGE_2_ synthesis pathway in microsomes

**Fig. 9:**
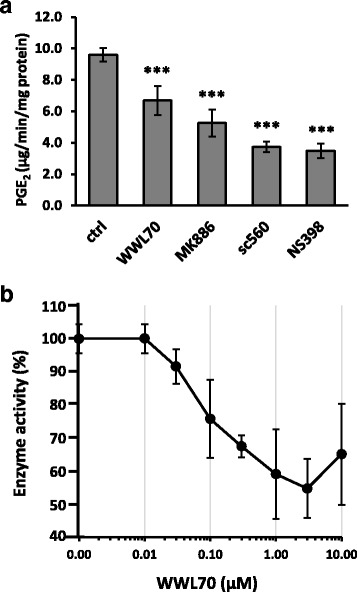
WWL70 inhibited microsomal PGE_2_ biosynthetic pathway. Microsome from LPS-activated BV2 cells was pre-incubated with WWL70 (3 μM), the mPGES-1 inhibitor MK886 (3 μM), the COX-1 inhibitor sc-560 (1 μM), or the COX-2 inhibitor NS398 (10 μM) for 5 min, then incubated with 10 μM AA for 1 min at 23 °C. The reaction mixture after incubation was employed PGE_2_ EIA (**a**). Dose-dependent inhibition of WWL70 was examined to determine its IC_50_ on PGE_2_ synthesis (**b**). Data are presented as mean ± SD (*n* = 3–6). *Triple asterisks* denote *p* < 0.001, respectively

To examine whether WWL70 directly inhibits the activity of COX enzymes, COX activity was assayed in vitro in the presence of WWL70 using ovine COX-1 and human COX-2 recombinant proteins. WWL70 did not have any effect on the activities of COX-1 or COX-2, while their activities were completely blocked by the selective COX-1 and COX-2 inhibitors SC560 and Dup697, respectively (Additional file 2: Figure S2). Since PGE_2_ biosynthetic enzymes are mainly localized in microsomes, we examined PGE_2_ production activity in microsome fraction of BV2 cells activated by LPS for 8 h. Microsome fraction was incubated with 10 μM AA for 1 min, followed by measurement of PGE_2_ (Fig. 9a). The enzyme activity was reduced to 60% by WWL70, whereas the mPGES-1 inhibitor MK886, the COX-1 inhibitor SC-560, and the COX-2 inhibitor NS398 COX-2 inhibitor reduced the enzymatic activity to 55, 39, and 36%, respectively. The dose-response study indicated that the IC_50_ of WWL70 to inhibit the PGE_2_ biosynthesis is about 100 nM (Fig. 9b), which is comparable to its *C*_50_ for ABHD6 inhibition (70 nM) [17].

### WWL70 suppressed the PGE_2_ production in primary microglia cells

**Fig. 10:**
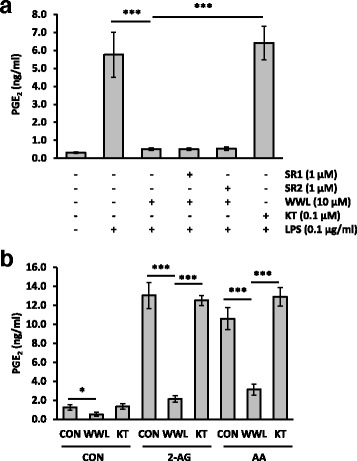
WWL70 inhibited PGE_2_ in primary microglia. PGE_2_ EIA of media from primary microglia. **a** Cells were treated with LPS for 18 h in the presence or absence of WWL70, KT182, or CB1 or CB2 receptor antagonist. **b** Microglia were treated with LPS for 18 h before media were replaced with DMEM containing WWL70 (10 μM), KT182 (0.1 μM), AA (10 μM), or 2-AG (10 μM) and incubated for 30 min. The data are represented as mean ± SD (*n* = 3). *Single* and *triple asterisks* denote *p* < 0.05 and 0.001, respectively

The effect of WWL70 on PGE_2_ production was also examined in rat primary microglia cells. Consistent with the results obtained in BV2 cells, overnight WWL70 treatment abolished PGE_2_ production in LPS-activated microglia, whereas KT182 did not have any inhibitory effects (Fig. 10a). The addition of CB1 and CB2 receptor antagonists did not reverse the inhibitory effect of WWL70 on PGE_2_, suggesting that the effect of WWL70 was not mediated by cannabinoid receptor activation. Primary microglia also replicated the effect in BV2 cells of short-term drug treatment (30 min) on the reduction of PGE_2_ production in LPS-stimulated cells supplemented with either 2-AG or AA (Fig. 10b). Cells were treated with LPS for 18 h and then switched to the medium containing WWL70 or KT182 for 30 min prior to PGE_2_ EIA. PGE_2_ production was enhanced approximately tenfold in the presence of either 2-AG or AA, indicating that the exogenous 2-AG and AA were metabolized to PGE_2_. Again, WWL70 but not KT182 inhibited PGE_2_ production.

### WWL70 suppressed PGE_2_ production in EAE mouse brain

**Fig. 11:**
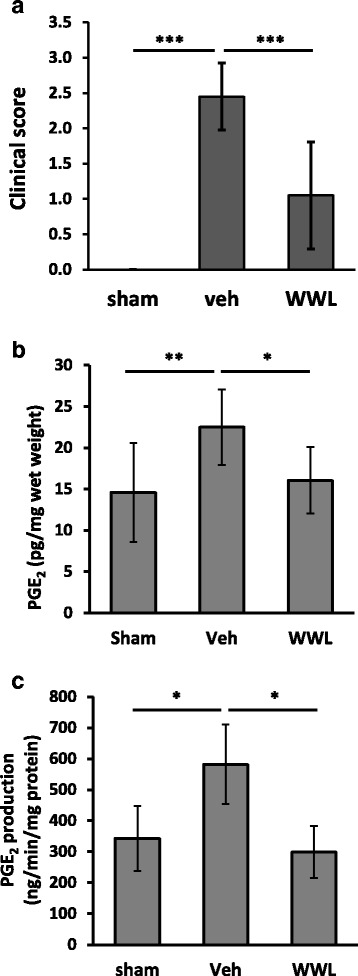
WWL70 treatment attenuated PGE_2_ production in EAE mouse brain. EAE mouse model was induced by immunization with the MOG_35–55_ peptide. Intraperitoneal injection of WWL70 was started at the disease onset and then once a day until the end of experiment. The clinical scores at day 21 in EAE mice in vehicle and drug-treated groups were shown (**a**). Mouse forebrain was dissected and used for measuring PGE_2_ concentration (*n* = 10–11) (**b**). Microsome fraction prepared from the brain tissue was applied to PGE_2_ synthetic activity assay (*n* = 4–5) (**c**). Data are represented as mean ± SD. *Single*, *double*, and *triple asterisks* denote *p* < 0.05, 0.01, and 0.001, respectively

We have recently reported that WWL70 reduces microglia activation, demyelination, and axonal injury and ameliorates clinical symptoms in the EAE mouse model [23]. To determine whether WWL70 could also inhibit PGE_2_ production in vivo, we examined EAE mouse brain tissue at day 21 post-immunization, at that time point, the clinical scores are significantly reduced (Fig. 11a). The levels of PGE_2_ in the EAE mice brain tissues increased by 1.5-fold compared to the control mice (Fig. 11b), which are similar to the previous study [31]. Treatment with WWL70 reversed the increased production of PGE_2_ (Fig. 11b). Consistently, the PGE_2_ biosynthetic activity in microsome fraction from the brain tissue was also increased in the EAE-vehicle group and reduced by WWL70 treatment (Fig. 11c). These results suggest that the reduced PGE_2_ production may contribute to the anti-inflammatory effect of WWL70 in the EAE mouse model.

## Discussion

WWL70, an ABHD6 selective inhibitor, possesses anti-inflammatory and neuroprotective effects in LPS-induced brain inflammation, traumatic brain injury, and multiple sclerosis [18, 22, 23]. Although ABHD6 is expressed in microglia and macrophages, and WWL70 can suppress microglia/macrophage activation, whether ABHD6 is the primary target for the action of WWL70 remains elusive. In this study, we found that WWL70 treatment inhibited 2-AG hydrolysis and increased the intracellular levels of 2-AG. WWL70 also significantly reduced the production of PGE_2_ in LPS-activated BV2 and primary microglia cells. However, the increased PGE_2_ production was not affected by another newly developed ABHD6 inhibitor KT182, and the inhibitory effect of WWL70 was also observed in BV2 cells with ABHD6 knockdown. These results suggest that the inhibitory effect of WWL70 on PGE_2_ production in LPS-activated microglia is not dependent on its inhibition of ABHD6 activity. Treatment with WWL70 reduced the expression of both COX2 and mPGES, the metabolic enzymes crucial for PGE_2_ production from AA. Furthermore, WWL70 directly inhibited the enzymatic activity of microsomal PGE_2_ biosynthesis.

It has recently been demonstrated that the hydrolysis of 2-AG by MAGL contributes significantly to the brain levels of AA [14]. Inhibition or deletion of MAGL has therapeutic effects in animal models of Parkinson’s disease [14] and Alzheimer’s disease [32] by blocking AA and PG production, but not by cannabinoid-receptor-mediated signaling. These recent findings highlight alternative therapeutic mechanisms of eCB-hydrolyzing enzyme inhibitors. Although MAGL is the primary enzyme for 2-AG hydrolysis in the brain, MAGL seems to play a minor role in microglial cells and macrophages because of its low expression [18, 19]. Instead, ABHD6 is one of the major hydrolyzing enzymes in macrophages as ABHD6 inhibition caused a twofold increase in 2-AG concentration [18]. We found that the increase in 2-AG by WWL70 was relatively mild in BV2 cells (Fig. 1c), possibly because the maximal inhibition by 10 μM of WWL70 only accounts for 40% of the total 2-AG hydrolysis (Fig. 1a). This result suggests that eCB signal potentiation by increased 2-AG may not be a major factor to the anti-inflammatory effect of WWL70 in microglia.

It is of note that the exogenously added 2-AG increased COX-2 protein expression in LPS-activated BV2 cells, whereas the commonly used synthetic CB receptor agonist WIN55,212-2 had an opposite effect on COX-2 expression (Fig. 3a). The increase of COX-2 expression by 2-AG is unlikely due to the activation of CB receptors. Similarly, AEA that is also metabolized to AA, or AA itself, increased the expression of COX-2 in LPS-activated BV2 cells, whereas 2-arachidonyl glyceryl ether (Noladin ether), an unhydrolyzable eCB ligand [33], had no effect. These results indicate that addition of AEA and 2-AG can increase AA production and facilitate eicosanoid signaling in reactive microglia through the increased expression of COX-2. Microsomal PGES1 and PGES2 were also increased in LPS-activated microglia and further potentiated by 2-AG. WWL70 suppressed COX-2 and mPGES1/2 expression in the absence or presence of 2-AG (Fig. 4). Taken together, these results suggest that the suppression of the metabolic enzymes to generate PGE_2_ contributes to the anti-inflammatory effects of WWL70.

**Fig. 12:**
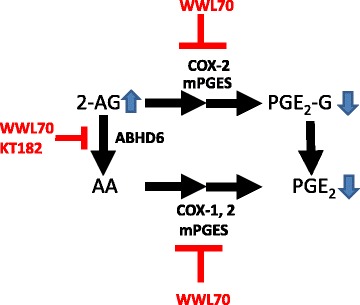
Potential actions of WWL70 on PGE_2_ and PGE_2_-G production in microglia. 2-AG is mainly metabolized to arachidonic acid, which in turn converts to PGE_2_ by catalysis of COX-1/2 and mPGESs. WWL70 inhibits ABHD6 thereby increasing the levels of 2-AG and reduces the production of PGE_2_ by inhibiting PGE_2_ synthetic enzymes. 2-AG can also be oxygenated by COX-2 and then converted to PGE_2_ glycerol ester by mPGESs, which is also inhibited by WWL70

Recently, it has been reported that WWL70 in mouse macrophages modulates the production of various PGs including PG-glycerol esters (PG-Gs) [18], which are the oxygenation product of 2-AG by COX-2 and PG synthases [34]. These results suggest that WWL70 also modulates the PGE_2_-G metabolism by increasing the availability of 2-AG and alteration of COX-2 and mPGES. Consistently, we also found that the generation of PGE_2_, as well as PGE_2_-G, in LPS-activated microglia was enhanced by the addition of 2-AG, and this increase was significantly inhibited by WWL70 (Fig. 8). Several studies have shown that PGE_2_-G triggers a pro-inflammatory response [35–37], while PGD_2_-G has an anti-inflammatory effect [18]. It is possible that WWL70 interferes with the PGE_2_-G synthetic pathway, which in turn promotes the production of PGD_2_-G by the increased availability of 2-AG (Fig. 12). It is still unclear how various PG-Gs can modulate the pro- and anti-inflammatory responses and how they differ from the actions of PGs, such as PGD_2_, PGE_2_, and PGF_1α_.

WWL123, a derivative of WWL70, has been shown to be protective in the animal model of epileptic seizures [24]. We have recently shown that the beneficial effect of WWL70 in the mouse model of traumatic brain injury [22] is at least in part due to its activation of CB1 and CB2 receptors, whereas in the experimental autoimmune encephalomyelitis mouse model, the therapeutic effect of WWL70 is chiefly mediated by the activation of CB2 receptors, as assessed by the use of the selective CB2 receptor antagonist and the CB2 receptor knockout mice [23]. Our present study suggests that the beneficial effect of WWL70 in these animal models might be also due to the reduced expression of COX-2 and PGES and the inhibition of the enzymatic activity for PGE_2_ production. Indeed, we found that the IC_50_ of WWL70 for PGE_2_ synthesis is about 100 nM, which is comparable to its inhibition on ABHD6 (70 nM) reported previously [17]. It has been demonstrated that mPGES1 is localized in microglia in the EAE mouse spinal cord and that PGE_2_ is a critical lipid mediator in microglia causing neuroinflammation through activation of EP_2_ receptor [38]. In LPS-activated BV2 microglia, we observed that the expression of EP_2_ receptor was upregulated and reduced by WWL70 treatment (data not shown). It is likely that the beneficial effect of WWL70 is also linked to the signaling mediated by EP_2_ receptor or other PGE_2_ receptors.

## Conclusions

Our data suggest that WWL70 can enhance the cannabinoid signaling and reduction of the eicosanoid signaling in microglia and may serve as an inhibitory modulator for the metabolism from 2-AG to PGE_2_ (Fig. 12). Thus, we speculate that WWL70 might be effective for the treatment of EAE and other neuroinflammatory and neurodegenerative diseases in which activation of the COX-2-PGES-PGE_2_ axis is a major contributor to the pathogenic mechanisms.

## Additional files

Additional file 1: Figure S1. Conversion of 2-AG to AA in membrane fraction. Membrane fraction from BV2 cells was incubated with 100 μM of 2-AG-d8 for 0, 1, or 5 min at 37 °C. AA-d8 extracted with acetonitrile was applied to LC-MS/MS. Mass spectrograms for the MRM transition from *m*/*z* 312 to *m*/*z* 93 is shown at three time points. Transition peaks of each time point are shown in inset represented as mean ± SD (*n* = 3). (PPTX 53 kb)
Additional file 2: Figure S2. Effect of WWL70 in recombinant COX assay. Ovine recombinant COX-1 (A) or human recombinant COX-2 (B) was pre-incubated in 0.1 M Tris-HCl (pH 8.0) and EDTA (1 mM) buffer with WWL70 (10 μM), SC-560 (0.33 μM), or Dup-697 (0.3 μM) for 5 min at 27 °C. Then, 10 μM of AA was added and incubated for 1 min at 27 °C. The reaction mixture was applied for prostaglandins EIA to measure total amount of prostaglandins. (PPTX 42 kb)

## Abbreviations

2-AG: 2-Arachidonoylglycerol
AA: Arachidonic acid
ABHD6: α/β-Hydrolase domain 6
ABPP: Activity-based protein profiling
AEA: N-arachidonoylethanolamine or anandamide
CB: Cannabinoid
CNS: Central nervous system
COX-2: Cyclooxygenase-2
EAE: Experimental autoimmune encephalomyelitis
eCB: Endocannabinoid
EIA: Enzyme-linked immunoassay
LC-MS/MS: Liquid chromatography coupled with tandem mass spectrometry
LPS: Lipopolysaccharide
MAGL: Monoacylglycerol lipase
MOG: Myelin oligodendrocyte glycoprotein
mPGES: Microsomal prostaglandin E2 synthase
Noladin ether: 2-Arachidonyl glyceryl ether
PG-Gs: Prostaglandin glycerol esters
PGs: Prostaglandins
SR1: SR141716A
SR2: SR144528
TFA: Trifluoroacetic acid
WIN: WIN55,212-2
