# Supplementary figures and images for: WWL70 attenuates PGE2 production derived from 2-arachidonoylglycerol in microglia by ABHD6-independent mechanism
Source: J Neuroinflammation. 2017 Jan 10;14:7. doi: 10.1186/s12974-016-0783-4 (PMC5234251; doi:10.1186/s12974-016-0783-4)

## Slide 1
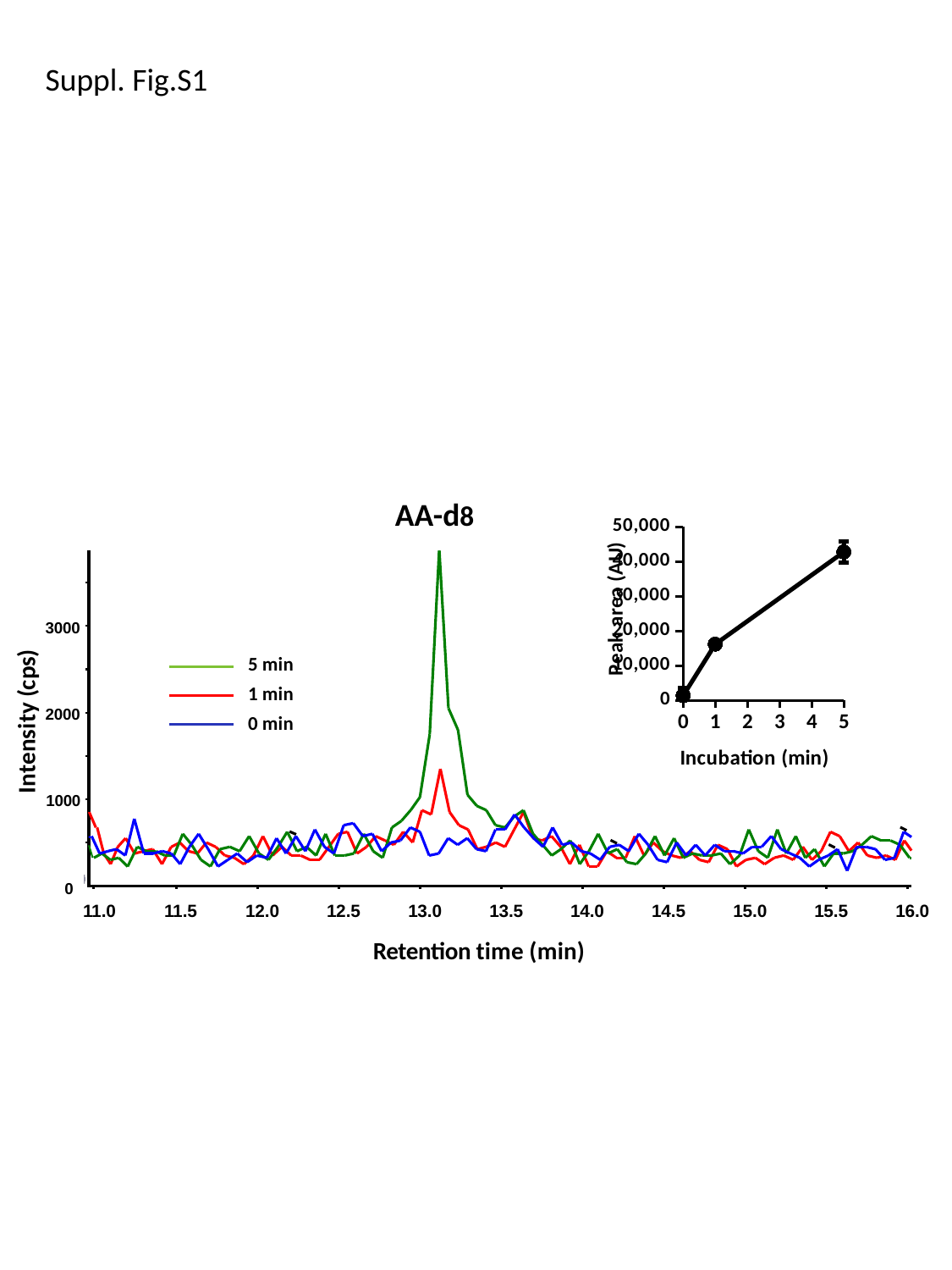

Suppl. Fig.S1
AA-d8
### Chart
| Category | d8-AA |
|---|---|
Intensity (cps)
3000
5 min
1 min
2000
0 min
1000
0
11.0
11.5
12.0
12.5
13.0
13.5
14.0
14.5
15.0
15.5
16.0
Retention time (min)

Supplement: Additional file 1: Figure S1. — Conversion of 2-AG to AA in membrane fraction. Membrane fraction from BV2 cells was incubated with 100 μM of 2-AG-d8 for 0, 1, or 5 min at 37 °C. AA-d8 extracted with acetonitrile was applied to LC-MS/MS. Mass spectrograms for the MRM transition from m/z 312 to m/z 93 is shown at three time points. Transition peaks of each time point are shown in inset represented as mean ± SD (n = 3). (PPTX 53 kb) [file 12974_2016_783_MOESM1_ESM.pptx]

## Slide 1
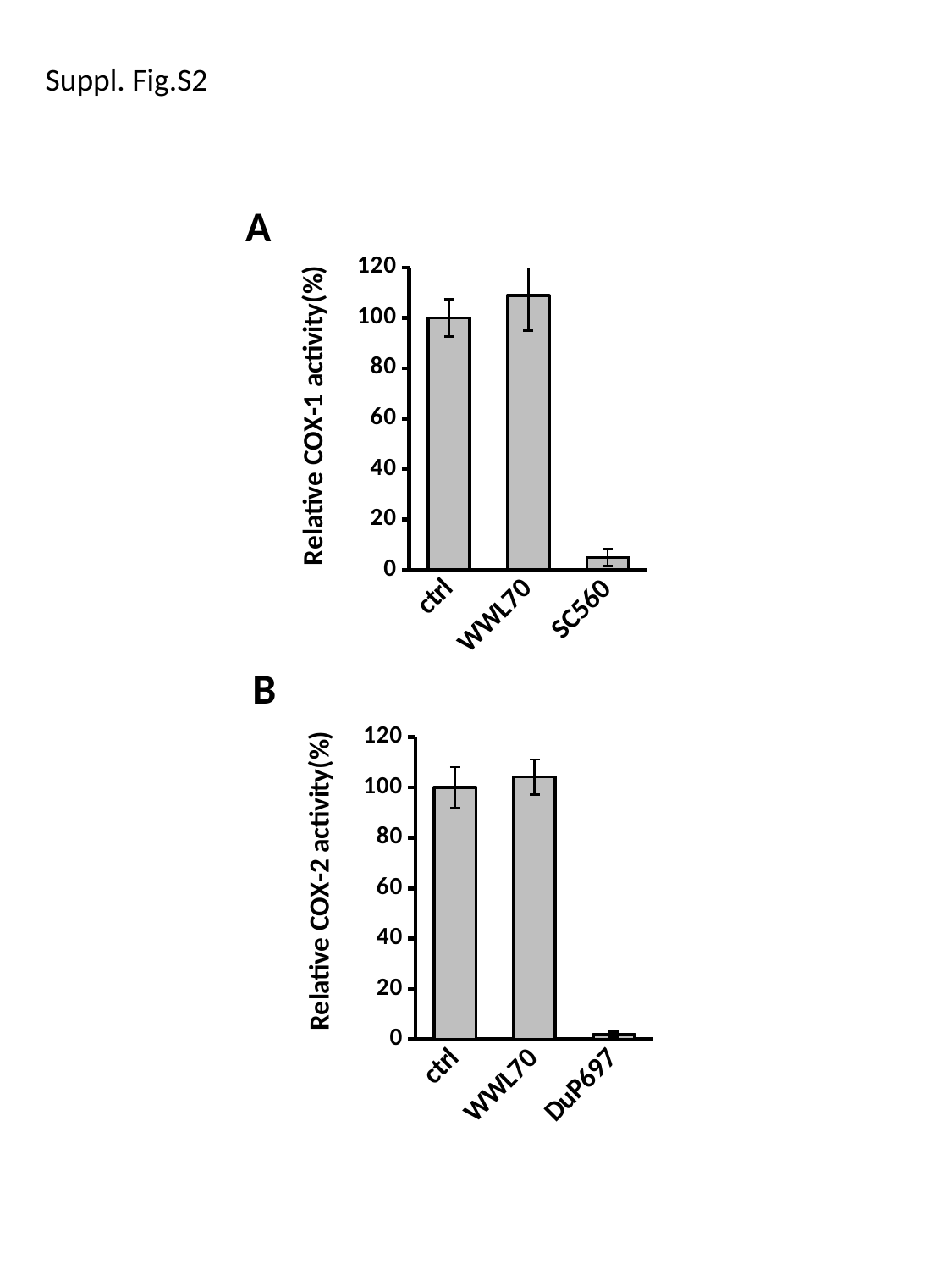

Suppl. Fig.S2
A
### Chart
| Category | |
|---|---|
| ctrl | 99.99995000002782 |
| WWL70 | 108.79856587898033 |
| SC560 | 4.912649863948371 |B
### Chart
| Category | |
|---|---|
| ctrl | 99.99999430519117 |
| WWL70 | 104.19167796549985 |
| DuP697 | 1.9832530483508628 |

Supplement: Additional file 2: Figure S2. — Effect of WWL70 in recombinant COX assay. Ovine recombinant COX-1 (A) or human recombinant COX-2 (B) was pre-incubated in 0.1 M Tris-HCl (pH 8.0) and EDTA (1 mM) buffer with WWL70 (10 μM), SC-560 (0.33 μM), or Dup-697 (0.3 μM) for 5 min at 27 °C. Then, 10 μM of AA was added and incubated for 1 min at 27 °C. The reaction mixture was applied for prostaglandins EIA to measure total amount of prostaglandins. (PPTX 42 kb) [file 12974_2016_783_MOESM2_ESM.pptx]
